# A validation of the Croatian version of Zarit Burden Interview and clinical predictors of caregiver burden in informal caregivers of patients with dementia: a cross-sectional study

**DOI:** 10.3325/cmj.2020.61.527

**Published:** 2020-12

**Authors:** Jelena Lucijanić, Ksenija Baždarić, Dina Librenjak, Marko Lucijanić, Miroslav Hanževački, Vesna Jureša

**Affiliations:** 1 Health Care Center Zagreb-West, Zagreb, Croatia; 2 Department of Medical Informatics, Faculty of Medicine, University of Rijeka, Rijeka, Croatia; 3 Sveti Ivan Psychiatric Hospital, Zagreb, Croatia; 4 Hematology Department, Dubrava University Hospital, Zagreb, Croatia; 5 University of Zagreb School of Medicine, Zagreb, Croatia

## Abstract

**Aim:**

To validate the Croatian version of the Zarit Burden Interview (ZBI) and to investigate the predictors of perceived burden.

**Methods:**

This cross-sectional study involved 131 dyads of one informal caregiver family member and one patient with dementia visiting primary care practices (Health Care Center Zagreb-West; 10/2017-9/2018). Patient-related data were collected with the Mini-Mental-State-Examination, Barthel-index, and Neuropsychiatric-Inventory-Questionnaire (NPI-Q); caregiver-related data with the ZBI, and general information on caregivers and patients with a structured questionnaire. Principal-axis-factoring with varimax-rotation was used for factor analysis.

**Results:**

The caregivers' mean age was 62.1 ± 13 years. They were mostly women (67.9%) and patients' children (51.1%). Four dimensions of ZBI corresponding to personal strain, frustration, embarrassment, and guilt were assessed and explained 56% variance of burden. Internal consistency of ZBI (α = 0.87) and its dimensions (α_1_ = 0.88, α_2_ = 0.83, α_3_ = 0.72, α_4_ = 0.75) was good. Stronger cognitive and functional impairment of patients was associated only with personal strain, whereas more pronounced neuropsychiatric symptoms and the need for daily care were associated with more dimensions. Longer caregiver education suppressed embarrassment and promoted guilt. Guilt was higher in younger caregivers, caregivers of female patients, patients' children, and non-retired caregivers. In multivariate analysis significant predictors of higher overall burden were male sex of the patient, higher NPI-Q, the need for daily-care services, shorter duration of caregiving, non-spouse relationship, higher number of hours caring per-week, and anxious-depressive symptoms in a caregiver.

**Conclusion:**

The Croatian version of ZBI is reliable and valid. Our data confirm that ZBI is a multidimensional construct. Caregivers may benefit from individually tailored interventions.

Dementia is an increasing health care problem associated with population aging (1-3). There are 8.9 million persons worldwide caring for patients with dementia older than 50 years (4). Caregiving for a family member with dementia substantially affects all aspects of informal caregivers' lives and demands lifestyle reorganization and adaptation. Usually, one family member becomes a dominant caregiver, devoting three quarters of a day to caregiving tasks, an amount of time that increases with disease progression (5). Caregivers often neglect their own needs and health problems and become increasingly exposed to physical, emotional, financial, and other loads, all of which can be assembled under the term caregiver burden (6). Patients with dementia frequently experience neuropsychiatric symptoms, which become an increasingly difficult problem, often worse than cognitive deterioration itself (7-9). These symptoms can lead to an inability of the informal caregivers to care for patients within her or his own family and increase the perceived caregiving burden.

The most commonly used tool for the assessment of caregiver burden is the Zarit Burden Interview (ZBI) (10). The original 29-item version was shortened to a 22-item version, which is currently the most widely used interview form. Several author groups showed that ZBI was a multidimensional construct, and that caregivers with the same total score might be differently affected by different aspects of burden (11-13). In addition, different burden dimensions might be differently affected by caregiver-related factors such as age, socio-economic factors, family relationship, availability of social support, etc (14-16). The most notable patient-related factors that affect caregiver burden are the presence of neuropsychiatric symptoms (especially irritability, agitation, sleep disorders, anxiousness, apathy, and delusions) and loss of cognitive function (17-20). These considerations have important implications for the planning of appropriate caregiver-oriented interventions.

The number of patients with dementia in Croatia ranges from 67 000 (21) to 85 000 (22) (estimates from 2013 and 2010, respectively), approximately 15 000 out of whom reside in the wider Zagreb area. Due to population aging and migrations, these numbers are probably increasing. However, there is currently no official registry of patients with dementia or informal caregivers in Croatia that would provide a direct insight into the real magnitude of the problem. The population of informal caregivers of patients with dementia in Croatia has not been extensively studied so far. It was shown that a high proportion of caregivers suffer from anxious and depressive symptoms (23). In addition, in comparison with professionals, informal caregivers were more anxious and depressive, especially if they were of older age and lived in the same household with the patient (24). Caregiving burden was identified as a contributor to the satisfaction with social support (25).

There is currently no version of ZBI questionnaire validated for the Croatian population. Thus, the aims of our study were to validate the Croatian version of the ZBI, to evaluate the validity and internal consistency of the questionnaire, and to assess the relationship of caregivers’ and patients’ characteristics with total and different aspects of caregiver burden.

## Methods

### Participants and setting

The study was conducted in 60 family medicine practices in the Health Care Center Zagreb-West from October 2017 to September 2018.

Among insured persons, we identified dyads consisting of a non-institutionalized patient with dementia (having International Classification of Diseases [ICD-10] codes F00, F01, F02, F03, or G30 determined by either a neurology or psychiatry specialist) and a family member being the dominant informal caregiver. The study did not include dyads consisting of a patient and an informal caregiver who was not a family member (neighbor, friend, paid informal caregiver). In addition, patients who were not diagnosed by either neurology or psychiatry specialists were not included. Dementia subtypes that were present were Alzheimer’s disease (76 patients), vascular dementia (47 patients), Parkinson’s disease dementia (6 patients), Lewy body dementia (1 patient), and frontotemporal dementia (1 patient). The patients with non-Alzheimer’s disease and non-vascular dementia were grouped together.

The study aimed to include 130 dyads in line with previous studies (12), as this was estimated as a sufficient sample size to perform ZBI validation and reproduce previously reported associations.

### Procedure

All family practitioners working in the Health Care Center Zagreb-West were approached with a request to screen for patients with dementia in their practices and to invite the patients' family members over the telephone to participate in the study.

The families willing to participate were contacted again by an investigator to arrange a meeting with the caregiver and patient. The meeting was arranged either in the caregiver's household or in the family doctor’s office. The caregivers (and patients if applicable) were informed in detail about the study aims and methods. After having signed a written informed consent, they completed a set of structured questionnaires. Caregivers were interviewed separately to more easily answer the questions.

### Inventory

*Zarit Burden Interview* (10) is a self-report tool consisting of 22 Likert-scale items (0 – never to 4 – almost always) used to assess the level of caregiver burden. The maximum score is 88 points, with a higher score representing a higher perceived caregiver burden. The originally proposed burden levels are as follows: absent to mild burden (0-20 points), mild to moderate burden (21-40 points), moderate to severe burden (41-60 points), and severe burden (61-88 points).

*Mini Mental State Examination (MMSE*) (26,27) was administered to the patient to assess the level of cognitive impairment. The maximum score of this 11-item tool is 30 points, calculated by adding up scores on individual items. A lower score represents a higher level of cognitive impairment.

*Barthel index* (28) was administered to the caregiver to assess the patients' level of functional impairment. The maximum score of this 10-item tool is 100 points, calculated by adding up scores on individual items. A lower score represents a higher level of functional impairment.

*Neuropsychiatric Inventory Questionnaire (NPI-Q)* (29) was administered to the caregiver to assess the presence and severity of neuropsychiatric symptoms in the patient. It investigates 12 domains, with each domain being related to the severity and frequency of neuropsychiatric symptoms. Domain scores are calculated by multiplying Likert-scale scores on severity (1 – mild to 3 – severe) and frequency (1 – rarely to 4 – very frequently), and the total NPI-Q score is calculated by adding up individual domain scores. NPI-Q also estimates the severity of distress associated with individual domains on a Likert-score scale (0 – none to 5 – very strong or pronounced), and total NPI-Q distress score is calculated by adding up individual domain scores. A higher total NPI-Q score and total NPI-Q distress score represent more pronounced neuropsychiatric symptoms and associated distress, respectively.

*A structured questionnaire including general questions about the caregiver and the patient*. Caregiver-related data were age, sex, years of education, employment status, relationship with the patient, length of caregiving, living in the same household with the patient, the need for daily care services, hours dedicated per week, hours of someone else’s help per week, smoking status, and morbidities. Patient-related data were age, sex, years of education, type of dementia, duration of dementia, and comorbidities. The assessed morbidities/comorbidities were arterial hypertension, diabetes mellitus, malignant disease, rheumatic symptoms requiring therapy, asthma/chronic obstructive lung disease in both the caregiver and patient, and anxious-depressive symptoms requiring therapy in the caregiver.

The MMSE was administered first, after which the patient was escorted to a nearby room, accompanied either by a nurse (if the visit was done in the Healthcare Center) or by other family members (if the visit was done in the household). Then the caregiver was administered the structured questionnaire, followed by ZBI, NPI-Q, and Barthel index.

The study was approved by the Health Care Center Zagreb-West and University of Zagreb School of Medicine Institutional Review Boards. Appropriate written informed consents were obtained from each caregiver and from the patients whenever clinically possible; otherwise the caregiver gave additional consent for the patient.

### Statistical methods

Categorical variables are presented as frequencies and relative frequencies. The normality of distribution of numerical variables was tested with the Kolmogorov-Smirnov test. The age variable was the only normally distributed variable and is presented as mean ± standard deviation, whereas other numerical variables were non-normally distributed and are presented as median and interquartile range (IQR).

After the Kaiser-Meyer-Olkin measure of sampling adequacy and the Bartlett test indicated that factor analysis can be performed, principal axis factoring with varimax rotation was conducted. Reliability was expressed with Cronbach alpha coefficient of internal consistency.

ZBI scores were compared between the groups with the Mann-Whitney U test or Kruskal-Wallis one way analysis of variance (ANOVA) test with the *post-hoc* test by Conover. Correlation with numerical variables was assessed with the Spearman coefficient of correlation (ρ).

Logistic regression was used for multivariate analysis of predictors of higher perceived burden where ZBI was the dependent variable dichotomized at 40 points (higher score representing moderate to severe and severe burden). Age and sex of the caregiver, age and sex of the patient, MMSE score, Barthel index score, total NPI-Q score, the need for daily care services, and all other variables that were univariately associated with overall ZBI score or any of its dimensions were included into the variable selection process via backward method (inclusion criterion *P* < 0.15, exclusion criterion *P* < 0.2). *P* values <0.05 were considered significant. The analyses were conducted with MedCalc version 19.0.4. (MedCalc Software bvba, Ostend, Belgium) and SPSS trial version (IBM, Armonk, NY, USA).

## Results

### Response rate

**Figure 1:**
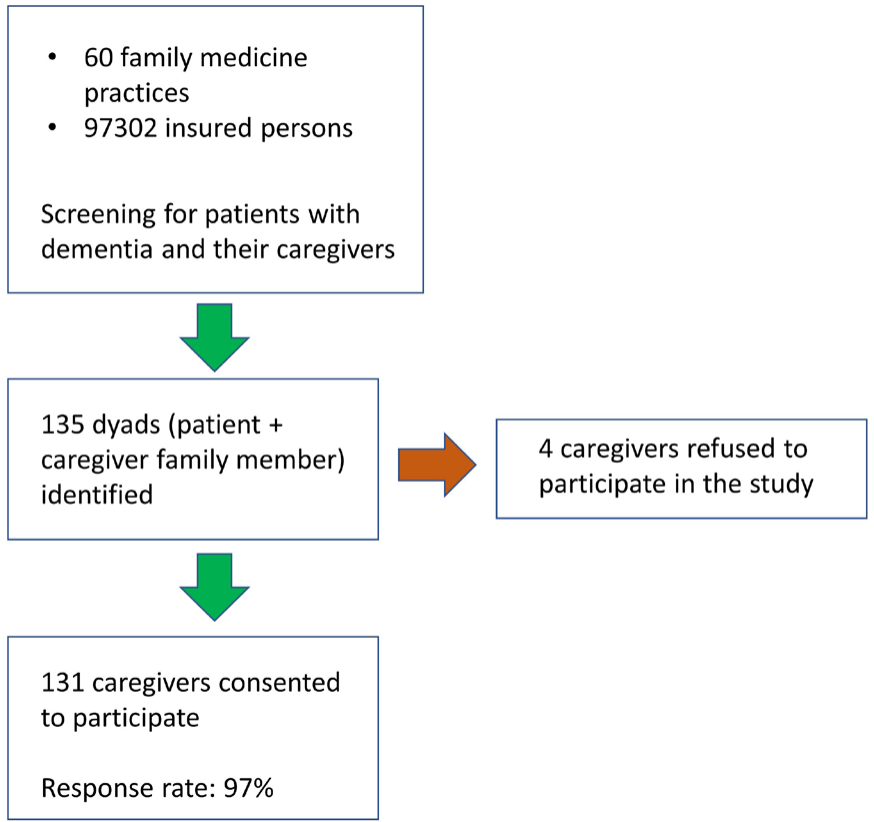
Flowchart of the study.

Among 97 302 insured persons in 60 family medicine practices in the Health Care Center Zagreb-West, we identified 135 dyads consisting of a non-institutionalized patient with dementia and a family member being the dominant informal caregiver. A total of 4 (3%) caregivers did not consent to participate, whereas 131/135 (97%) dyads of caregivers and patients with dementia were included in the study (Figure 1).

### General characteristics

The mean age was 62.1 ± 13 years for caregivers and 79.4 ± 7.1 for patients with dementia. The majority of caregivers (89/131 or 67.9%) and patients (92/131 or 70.2%) were women. Most of caregivers were patients' children (67/131 or 51.1%), followed by spouses (51/131 or 38.9%). Most of patients had Alzheimer’s dementia (76/131 or 58%), with the median MMSE score of 15, Barthel index score of 85, and mean NPI-Q score of 26, corresponding to moderate dementia severity, the absence of functional dependency, and the presence of neuropsychiatric symptoms, respectively.

### Construct validity and reliability

Internal consistency of the whole ZBI scale was high (Cronbach α coefficient for the overall ZBI score was 0.89). Factor analysis was appropriate as Kaiser-Meyer-Olkin measure of the sampling adequacy was 0.84 and the Bartlett test of sphericity was significant (*P* < 0.001).

Principal axis factoring with varimax rotation resulted in an extraction of 5 factors with eigenvalue >1, which accounted for 61% of total variance. The fifth factor was not interpretable as it overlapped with different items. We further conducted the same factor analysis with the extraction of 4 factors, which accounted for 56% of total variance. Structure matrix (correlations of each item with the extracted dimensions) for factor analysis with 4 factors is shown in Table 1.

**Table 1:** Pattern matrix of the exploratory factor analysis*

	Zarit Burden Interview (ZBI) factor loadings			
Items	F1: personal strain	F2: frustration	F3: embarrassment	F4: guilt
**ZBI1:** Do you feel that your relative asks for more help than he/she needs?			0.70	
**ZBI2:** Do you feel that because of the time you spend with your relative you don’t have enough time for yourself?	0.79			
**ZBI3:** Do you feel stressed between caring for your relative and trying to meet other responsibilities?	0.59	0.40		
**ZBI4:** Do you feel embarrassed over your relative’s behavior?			0.61	
**ZBI5:** Do you feel angry when you are around your relative?		0.67		
**ZBI6:** Do you feel that your relative currently affects your relationship with other family members or friends in a negative way?		0.43	0.52	
**ZBI7:** Are you afraid of what the future holds for your relative?				
**ZBI8:** Do you feel your relative is dependent on you?	0.55			
**ZBI9:** Do you feel strained when you are around your relative?		0.72		
**ZBI10:** Do you feel your health has suffered because of your involvement with your relative?	0.56			
**ZBI11:** Do you feel that you don’t have as much privacy as you would like, because of your relative?	0.67			
**ZBI12:** Do you feel that your social life has suffered because you are caring for your relative?	0.76			
**ZBI13:** Do you feel uncomfortable about having friends over, because of your relative?			0.55	
**ZBI14:** Do you feel that your relative seems to expect you to take care of him/her as if you were the only one he/she could depend on?			0.42	
**ZBI15:** Do you feel you don’t have enough money to support your relative in addition to the rest of your expenses?				
**ZBI16:** Do you feel that you will be unable to take care of your relative much longer?		0.69		
**ZBI17:** Do you feel you have lost control of your life since your relative’s illness?	0.54			
**ZBI18:** Do you wish you could just leave the care of your relative to someone else?	0.37	0.48		
**ZBI19:** Do you feel uncertain about what you do about your relative?				
**ZBI20:** Do you feel you should be doing more for your relative?				0.69
**ZBI21:** Do you feel you could do a better job in caring for your relative?				0.72
**ZBI22:** Finally, do you feel that care for your relative is a burden for you?	0.60			

*Extraction method: principal axis factoring. Rotation method: varimax with Kaiser normalization.

The first factor accounted for 31% of variance (eigen = 6.94), included 9 items (2,3,8,10-12,17,18,22) with Cronbach α of 0.88, and may be interpreted as personal strain. The second factor accounted for 10% of variance (eigen = 2.06), included 6 items (3,5,6,9,16,18) with Cronbach α of 0.83, and may be interpreted as frustration. The third factor accounted for 9% of variance (eigen = 1.94), included 5 items (1,4,6,13,14) with Cronbach α of 0.72, and may be interpreted as embarrassment. The fourth factor accounted for 7% of variance (eigen = 1.44), included 2 items (20,21) with Cronbach α of 0.75, and may be interpreted as guilt. The items 7, 15, and 19 were not included in any of the extracted factors and the items 3, 6, and 18 had loadings on different factors.

All four factors were significantly correlated with the overall ZBI score, factor 1 and factor 2 being strongly correlated (ρ 0.9 and 0.82, respectively), factor 3 being moderately correlated (ρ 0.68), and factor 4 being weakly (ρ 0.24) correlated with the overall ZBI score.

### Zarit Burden Interview

The median overall ZBI score was 27 (IQR, 21-39), which corresponds to a mild to moderate perceived burden. According to cut-offs proposed by Zarit and Zarit (10), 31/131 (23.7%) caregivers had absent to mild burden, 31/131 (23.7%) mild to moderate burden, 23/131 (17.6%) moderate to severe burden, and 6/131 (4.6%) had severe burden. There was no difference in the overall ZBI score or in ZBI dimension scores between male and female caregivers (*P* > 0.05 for all comparisons) (Table 2).

**Table 2:** Overview of caregivers’ and patients’ characteristics and their relationship with the overall Zarit Burden Interview (ZBI) and factor scores

	Summary, n (%) or median (interquartile range) or mean ± standard deviation	Overall ZBI score	F1 personal strain	F2 frustration	F3 embarrassment	F4 guilt
Caregiver's age (years), mean ± standard deviation	62.1 ± 13	ρ = -0.05 *P* = 0.610^§^	ρ = -0.09 *P* = 0.301^§^	ρ = -0.01 *P* = 0.919^§^	ρ = -0.02 *P* = 0.865^§^	ρ = -0.21 *P* = 0.015^§^
Caregiver sex		Median	Median	Median	Median	Median
male	42/131 (32.1)	25	12.5	6	3.5	2
female	89/131 (67.9)	29 *P* = 0.315^‡^	15 *P* = 0.345^‡^	7 *P* = 0.545^‡^	5 *P* = 0.062^‡^	1 *P* = 0.257^‡^
Caregiver education (years)	12 (12-16)	ρ = -0.14 *P* = 0.113^§^	ρ = -0.09 *P* = 0.323^§^	ρ = -0.03 *P* = 0.725^§^	ρ = -0.23 *P* = 0.009^§^	ρ = 0.25 *P* = 0.004^§^
Caregiver employment status		Median	Median	Median	Median	Median
employed	58/131 (44.3)	28.5	15	6	4	2
unemployed	7/131 (5.3)	31	16	7	4	4
retired	66/131 (50.4)	27 *P* = 0.848^+^	13.5 *P* = 0.870^+^	6.5 *P* = 0.659^+^	5 *P* = 0.424^+^	0 *P* = 0.003^+^ (1 vs 3; 2 vs 3)*
Caregiver relationship with a patient		Median	Median	Median	Median	Median
spouse	51/131 (38.9)	27	12	6	4	0
children	67/131 (51.1)	32	16	6	5	2
other	13/131 (9.9)	26 *P* = 0.272^+^	15 *P* = 0.295^+^	6 *P* = 0.576^+^	5 *P* = 0.686^+^	2 *P* = 0.006^+^ (1 vs 2)
Living in the same household		Median	Median	Median	Median	Median
yes	112/131 (85.5)	28.5	15	6.5	5	1.5
no	19/131 (14.5)	19 *P* = 0.007^‡^	8 *P* = 0.030^‡^	5 *P* = 0.091^‡^	4 *P* = 0.105^‡^	2 *P* = 0.586^‡^
Length of caregiving (years)	2 (1-3)	ρ = 0.2 *P* = 0.024^§^	ρ = 0.3 *P* < 0.001^§^	ρ = 0.13 *P* = 0.155^§^	ρ = 0.02 *P* = 0.821^§^	ρ = 0.02 *P* = 0.865^§^
Hours dedicated per week	56 (35-75)	ρ = 0.27 *P* = 0.002^§^	ρ = 0.31 *P* < 0.001^§^	ρ = 0.15 *P* = 0.096^§^	ρ = 0.23 *P* = 0.009^§^	ρ = -0.04 *P* = 0.618^§^
Hours of help received per week	24 (1.5-49)	ρ = 0.2 *P* = 0.021^§^	ρ = 0.26 *P* = 0.003^§^	ρ = 0.11 *P* = 0.228^§^	ρ = -0.01 *P* = 0.955^§^	ρ = 0.18 *P* = 0.035^§^
Caregiver's anxious-depressive symptoms		Median	Median	Median	Median	Median
yes	24/131 (18.3)	36.5	17.5	7.5	5	1.5
no	107/131 (81.7)	27 *P* = 0.013^‡^	14 *P* = 0.037^‡^	6 *P* = 0.067^‡^	4 *P* = 0.082^‡^	2 *P* = 0.776^‡^
Patient age (years)	79.4 ± 7.1	ρ = 0.06 *P* = 0.518^§^	ρ = 0.06 *P* = 0.518^§^	ρ = 0.09 *P* = 0.286^§^	ρ = 0.06 *P* = 0.691^§^	ρ = 0.08 *P* = 0.382^§^
Patient's sex		Median	Median	Median	Median	Median
male	39/131 (29.8)	28	14	7	7	0
female	92/131 (70.2)	27 *P* = 0.791^‡^	15 *P* = 0.994^‡^	6 *P* = 0.884^‡^	4 *P* = 0.083^‡^	2 *P* = 0.021^‡^
Patient education (years)	12 (4-13)	ρ = -0.07 *P* = 0.434^§^	ρ = -0.11 *P* = 0.228^§^	ρ = -0.05 *P* = 0.571^§^	ρ = -0.04 *P* = 0.691^§^	ρ = 0 *P* = 0.964^§^
Type of dementia		Median	Median	Median	Median	Median
Alzheimer’s disease	76/131 (58)	27	16	6	4	2
vascular dementia	47/131 (35.9)	28	13	6	5	1
other	8/131 (6.1)	28.5 *P* = 0.893^+^	12.5 *P* = 0.664^+^	7 *P* = 0.947^+^	5.5 *P* = 0.690^+^	1 *P* = 0.852^+^
Duration of dementia (years)	4 (2-5)	ρ = 0.23 *P* = 0.009^§^	ρ = 0.3 *P* < 0.001^§^	ρ = 0.2 *P* = 0.024^§^	ρ = 0.05 *P* = 0.610^§^	ρ = 0.11 *P* = 0.219^§^
Hypertension in a patient with dementia		Median	Median	Median	Median	Median
yes	83/131 (63.4)	27	14	6	5	1
no	48/131 (36.6)	27.5 *P* = 0.895^‡^	15 *P* = 0.430^‡^	6.5 *P* = 0.682^‡^	3.5 *P* = 0.004^‡^	2 *P* = 0.382^‡^
Use of daily care services		Median	Median	Median	Median	Median
yes	33/131 (25.2)	36	18	8	5	3
no	98/131 (74.8)	26.5 *P* = 0.001^‡^	12 *P* = 0.001^‡^	6 *P* = 0.028^‡^	4 *P* = 0.059^‡^	1 *P* = 0.048^‡^
MMSE score	15 (9-20)	ρ = -0.16 *P* = 0.068^§^	ρ = -0.27 *P* = 0.002^§^	ρ = -0.09 *P* = 0.301^§^	ρ = 0.12 *P* = 0.169^§^	ρ = 0.1 *P* = 0.261^§^
Barthel index score	85 (65-100)	ρ = -0.23 *P* = 0.009^§^	ρ = -0.37 *P* < 0.001^§^	ρ = -0.09 *P* = 0.334^§^	ρ = 0.01 *P* = 0.874^§^	ρ = -0.05 *P* = 0.594^§^
NPI-Q score	26 (12-39)	ρ = 0.46 *P* < 0.001^§^	ρ = 0.5 *P* < 0.001^§^	ρ = 0.31 *P* < 0.001^§^	ρ = 0.33 *P* < 0.001^§^	ρ = 0.03 *P* = 0.717^§^
NPI-Q distress score	15 (7-23)	ρ = 0.53 *P* < 0.001^§^	ρ = 0.5 *P* < 0.001^§^	ρ = 0.39 *P* < 0.001^§^	ρ = 0.45 *P* < 0.001^§^	ρ = 0.01 *P* = 0.892^§^
ZBI score						
Overall	27 (21-39)	-	ρ = 0.9 *P* < 0.001^§^	ρ = 0.82 *P* < 0.001^§^	ρ = 0.68 *P* < 0.001^§^	ρ = 0.24 *P* = 0.007^§^
F1 personal strain	15 (8-21)	ρ = 0.9 *P* < 0.001^§^	-	ρ = 0.7 *P* < 0.001^§^	ρ = 0.44 *P* < 0.001^§^	ρ = 0.1 *P* = 0.265^§^
F2 frustration	6 (3-10)	ρ = 0.82 *P* < 0.001^§^	ρ = 0.7 *P* < 0.001^§^	-	ρ = 0.49 *P* < 0.001^§^	ρ = 0.21 *P* = 0.017^§^
F3 embarrassment	4 (3-8)	ρ = 0.68 *P* < 0.001^§^	ρ = 0.44 *P* < 0.001^§^	ρ = 0.49 *P* < 0.001^§^	-	ρ = 0.02 *P* = 0.838^§^
F4 guilt	2 (0-4)	ρ = 0.24 *P* = 0.007^§^	ρ = 0.1 *P* = 0.265^§^	ρ = 0.21 *P* = 0.017^§^	ρ = 0.02 *P* = 0.838^§^	-

*F – factor; MMSE – Mini Mental State Examination; NPI-Q – Neuropsychiatric Inventory Questionnaire.

^+^Kruskal-Wallis ANOVA test with post-hoc test by Conover.

^‡^Mann-Whitney U test.

^§^Spearman correlation.

In univariate analyses, the overall ZBI score was significantly higher in caregivers living in the same households as the patients (*P* = 0.007), caring for the patient for a longer time (*P* = 0.024), having more hours of caring per week (*P* = 0.002), needing more hours of someone else’s help per week (*P* = 0.021), having anxious-depressive symptoms requiring therapy (*P* = 0.013), caring for patients with a longer duration of dementia (*P* = 0.009), caring for patients having higher functional impairment as assessed by the Barthel index (*P* = 0.009), having more pronounced neuropsychiatric symptoms as assessed by NPI-Q score (*P* < 0.001), and needing daily care services (*P* = 0.001) (Table 2). In addition, a higher overall ZBI score was significantly moderately associated with higher distress caused by neuropsychiatric symptoms as assessed by total NPI-Q distress score (ρ = 0.53; *P* < 0.001).

Personal strain, frustration, and embarrassment were mutually significantly moderately correlated (ρ 0.44 to 0.70), whereas only embarrassment was significantly but weakly correlated with guilt (ρ = 0.21). Personal strain, frustration, and embarrassment were also significantly moderately correlated with total NPI-Q distress score (ρ 0.5, 0.39, and 0.45, respectively), whereas guilt was not significantly associated with NPI-Q distress (*P* = 0.892).

Higher scores on personal strain were associated with living in the same household as the patient (*P* = 0.030), longer duration of caregiving (*P* < 0.001), spending more hours of caring per week (*P* < 0.001), needing more hours of someone else’s help per week (*P* = 0.003), having anxious-depressive symptoms requiring therapy (*P* = 0.037), caring for patients with longer duration of dementia (*P* < 0.001), caring for patients having higher cognitive impairment as assessed by MMSE score (*P* = 0.002), higher functional impairment as assessed by the Barthel index (*P* < 0.001), more pronounced neuropsychiatric symptoms as assessed by NPI-Q score (*P* < 0.001), and the need for daily care services (*P* = 0.001).

Higher scores on frustration were associated with more pronounced neuropsychiatric symptoms as assessed by NPI-Q score (*P* < 0.001), longer duration of dementia (*P* = 0.024), and the need for daily care services (*P* = 0.028).

Higher scores on embarrassment were associated with fewer years of caregiver education (*P* = 0.009), spending more hours of caring per week (*P* = 0.009), arterial hypertension in the patient (*P* = 0.004), and more pronounced neuropsychiatric symptoms as assessed by NPI-Q score (*P* < 0.001).

Higher scores on guilt were associated with younger age (*P* = 0.015), higher number of years of caregiver education (*P* = 0.004), being unemployed or employed in comparison with being retired (*P* = 0.003), being a child in comparison with being the spouse of the patient (*P* = 0.006), caring for a female patient with dementia (*P* = 0.021), and the need for daily care services (*P* = 0.048).

### Multivariate predictors of higher perceived burden

In multivariate logistic regression with ZBI>40 (describing moderate to severe and severe burden) as a dependent variable we investigated an independent contribution of parameters that showed univariate associations with the overall ZBI or any of burden dimensions (described in detail in the Methods section). The model is shown in the Table 3.

**Table 3:** Logistic regression model for total Zarit Burden Interview score >40 (corresponding to moderate to severe perceived burden)*

	*P*	Odds ratio	95% confidence interval
Male sex of the caregiver	0.119	3.49	0.72-16.93
Caregiver's years of education	0.115	0.82	0.65-1.05
Caregiver's retirement	0.103	3.82	0.76-19.26
Caregiver's anxious-depressive symptoms	0.015	5.55	1.4-21.96
Length of caregiving (years)	0.028	0.64	0.43-0.95
Hours dedicated per week	0.007	1.04	1.01-1.07
Spouse relationship with the patient	<0.001	0.007	0-0.09
Use of daily care services	<0.001	11.25	2.8-45.21
Male sex of the patient	0.004	15.12	2.37-96.42
Barthel index	0.149	1.02	0.99-1.05
Total Neuropsychiatric Inventory Questionnaire score	0.008	1.05	1.01-1.08

*Overall *P* < 0.001; Nagelkerke R^2^ 50%.

Mutually independent predictors of higher overall ZBI score were male sex of the patient (OR 15.1; *P* = 0.004), higher NPI-Q score (OR 1.05; *P* = 0.008), the need for daily care services (OR 11.3; *P* < 0.001), shorter duration of caregiving (longer duration OR 0.64; *P* = 0.028), non-spouse relationship with the patient (spouse OR 0.01; *P* < 0.001), higher number of hours caring per week (OR 1.04; *P* = 0.007), and anxious-depressive symptoms requiring therapy in the caregiver (OR 5.6; *P* = 0.015), whereas male sex of the caregiver (OR 3.49; *P* = 0.119), the Barthel index (OR 1.02; *P* = 0.149), years of caregiver education (OR 0.82, *P* = 0.115), and retirement (OR 3.82; *P* = 0.103) were retained in a model but were rendered non-significant.

## Discussion

Our data show that ZBI is a multidimensional construct and that patients with the same overall burden might be differently affected by different burden aspects. In addition, our data confirm the reliability and validity of the Croatian version of ZBI. We identified four factors of the Croatian version of ZBI. In Italy, Chattat et al (30) obtained a 5-factor structure on a sample of caregivers (mostly patient’s children), where the items on factor 1 (personal strain) and 4 (guilt) were very similar to our factorial structure but the factors 2 and 3 did not correspond to ours. Some of the items (Factor 4: items 7 and 19) that were extracted in the study by Chattat et al (30) were not extracted in our study (were not relevant for creation of the factors), probably because of the translation or cultural differences. The first 3 factors in our study highly correlated with the overall ZBI score (ρ>0.65) and may be considered as one factor with 3 facets, while the fourth factor (guilt) weakly correlated with ZBI score and is to be considered independently. These results and conclusions correspond to the bifactorial structure proposed by Siegert et al (31) and other authors. The whole scale has high reliability, but as the factors vary across countries, we would recommend using it unidimensionally. There are several shortened versions of the scale, such as the 12-item version by Ballesteros et al (32), where 10 items correspond to the first factor in our study and 2 items (9,16) to the second factor. However, in our opinion omitting items that measure psychological aspects (items 4,5,6,7,13,14) and guilt, which is a very strong factor, does not increase the understanding of burden.

Significant differences in mean ZBI score were demonstrated between different European countries (33), with the highest burden in Estonia (39.7) and lowest in the Netherlands (26.5). A possible explanation is that Estonian caregivers spent a significantly higher number of hours assisting with daily activities and were younger, thus having additional obligations, such as child care and work commitments. The mean ZBI value in our study (30.6) is among the lowest reported values. This could be attributed to different enrollment criteria (the aforementioned study [33] enrolled caregivers whose patients were at risk for institutionalization in the next 6 months, whereas our study did not impose special conditions for enrollment). As much as 86% of the Croatian population declares themselves to be Catholics (34). Catholics might have lower perceived burden because they feel an obligation to care for their loved ones and will not seek help unless the burden creates a significant problem for them. Previous studies reported that countries with a higher level of religiosity had a lower degree of caregiving burden (35-37). Other cultural determinants, a differently organized system of social care, availability of social support (25), gross domestic product, and health care standards probably play a role in the observed differences as well. We believe that families that decide to care for their member with dementia at home are physically and mentally prepared for this task. If not, they can temporarily hospitalize the patient in a psychogeriatric department until a long-term solution is found, an option that might not be equally available in other countries.

Personal strain was the only factor significantly associated with higher cognitive impairment and higher functional dependency of the patient in contrast with other ZBI dimensions. In addition, it was associated with the intensity and duration of caregiving, the need for someone else’s help, and anxious-depressive symptoms requiring therapy in a caregiver. The second and third factor comprised of items concerning psychological aspects of caregiving corresponding to frustration and embarrassment, respectively. The fourth factor included the items 20 and 21 corresponding to guilt and is consistent with the results of previous studies that identified it and its weak correlations with the overall ZBI score and other ZBI dimensions (12,13,38,39). The need for daily care services was consistently associated with increased burden over all four dimensions (association being borderline significant for embarrassment). More pronounced neuropsychiatric symptoms moderately affected all recognized dimensions of burden except guilt. Caregiver education differently affected embarrassment and guilt, suppressing embarrassment and promoting guilt. Higher guilt was also associated with employment status (higher in employed and non-employed in comparison with retired caregivers), being the patient's child in comparison with being the spouse, and caring for a female patient with dementia. Negative correlation of guilt with age and association of higher guilt with being the patient's child has been recognized by other author groups as well (40). This phenomenon illustrates that caregiving experience differs for spouses and children caregivers, probably due to the fact that children caregivers usually need to balance between caring for their parent, work, and family responsibilities. However, our study is the first to associate guilt with caring for a female patient with dementia. Since women are those who traditionally assume the caregiving role in the family, other family members find it difficult to cope with their illness. Thus, they may feel a higher degree of guilt as they are not able to reciprocate the care they received and provide the level of care they think their female dependents deserve. Besides anxious-depressive symptoms requiring therapy in the caregiver and arterial hypertension in the patient, other comorbidities or smoking were not significantly associated with the perceived burden.

Multivariate analysis of predictors of the overall perceived burden revealed that it is more demanding to care for a male patient with dementia, as well as that patients' children experience higher degree of burden in comparison with the spouses, irrespective of the patient's sex, intensity and duration of caregiving, the need for daily care services, presence of neuropsychiatric symptoms in a patient, and anxious-depressive symptoms requiring therapy in a caregiver. It is interesting to note that in the context of all these variables, a longer duration of caregiving seems to ameliorate the burden, which is in contrast to unadjusted univariate associations of caregiving duration and higher burden. There was a consistent contribution of neuropsychiatric symptoms and the need for daily care services to both overall burden and most of its dimensions (association of the use of daily care services and embarrassment was borderline significant, whereas other dimensions were significantly associated). The use of daily care services may prolong the period when the patients can receive care within their family circle. The use of these services is a step preceding the patient's institutionalization and usually serves as a respite for the caregiver (41,42), who cannot leave the patient home alone when needing to leave the house. The use of daily care services represents help-seeking behavior, might indicate an impending breakdown of the caregiver (43), and was an important predictor of the caregiver burden in the current study.

There are several limitations of our study. First, the cross-sectional design prevented us from inferring any causal relationship between burden and investigated variables. Second, we made efforts to approach as many informal caregivers who are family members as possible through assessing the patients with particular ICD-10 diagnoses made by neurology or psychiatry specialists. Hence, a number of caregiver-patient dyads might have been missed if the patient with dementia was not evaluated by these specialists or the caregiver was not a family member. Third, the investigated caregiver group represents urban dwellers from the Croatian capital, which makes our results possibly not generalizable to rural areas or other Croatian regions. A larger national or regional-multinational study could provide a deeper insight into caregiver burden differences potentially associated with regional specificities.

Regardless of these limitations, our results have important implications for the recognition of variables associated with different aspects of caregiver burden and for the planning of therapeutic interventions aimed at burden amelioration. Family medicine practice is the optimal setting where caregivers can be timely recognized, their burden assessed, and targeted interventions implemented. Development of such measures is urgently needed.
